# Assessing Technical Skills in Talented Youth Athletes: A Systematic Review

**DOI:** 10.1007/s40279-020-01299-4

**Published:** 2020-06-03

**Authors:** Till Koopmann, Irene Faber, Joseph Baker, Jörg Schorer

**Affiliations:** 1 Institute of Sport Science, Carl von Ossietzky University Oldenburg, Ammerländer Heerstraße 114-118, 26111 Oldenburg, Germany; 2 International Table Tennis Federation, Lausanne, Switzerland; 3 School of Kinesiology and Health Science, York University, Toronto, Canada

## Abstract

**Background:**

Talent identification and development (TID) programs aim to identify players with the greatest potential for long-term success. Previous research suggests that the assessment of sport-specific technical skills is valuable for discriminating between more and less skilled individuals and/or for predicting future performance.

**Objective:**

This review aims to provide an overview on both the instruments used to assess sport-specific technical skills and their discriminatory, explanatory and/or predictive findings in the context of TID.

**Methods:**

Electronic searches were conducted in PubMed, Web of Knowledge, SPORTDiscus, SURF and Scopus (January 1990–October 2019). Search terms covered the areas of sport, technical skills assessment, performance, skill level and youth. In the end, 59 relevant studies were identified and evaluated.

**Results:**

The results highlight the widespread and important role of technical skills in TID; almost all studies (93%) reported discriminatory, explanatory and/or predictive benefits for the assessment of sport-specific technical skills. Analyzing and categorizing the number of assessment methods applied in the studies (*n* = 69) according to their method type (‘technique-related’ or ‘outcome-related’ variables) and method set-up (‘experimental’ or ‘competition’ data acquisition environment) indicated a clear tendency towards ‘outcome-related’ (73%) and ‘experimental’ (75%) assessment methods. We also found a strong overrepresentation of studies assessing cross-sectional data (75%) in soccer (53%) in male samples (74% of studies reporting subjects’ sex) from European countries (64%).

**Conclusions:**

On the one hand, our findings demonstrate the great capability of sport-specific technical skills assessments to discriminate different performance levels and predict future performance in TID activities. On the other hand, this review highlights the focus on ‘outcome-related’ and ‘experimental’ methods in specific populations and, consequently, the limited knowledge in other areas. Here, the application of ‘technique-related’ and ‘competition’ methods appears promising for adding new knowledge, especially in the light of technological advances.

## Key Points

**Table:** 

This systematic review summarizes and categorizes studies between the years 1990 and 2019 that investigated differences in sport-specific technical skills in young athletes from different skill or performance levels and/or the role of these skills in predicting later performance.
The included studies highlight the value of sport-specific technical skills in TID for discriminating different skill or performance levels and/or predicting later performance while depicting the narrow and limited knowledge we currently have in regard to different populations and various assessment methods.
These findings can be used by scientists to develop innovative study designs potentially providing new insights into TID, as well as by administrators and coaches to improve selection procedures and decision-making in TID contexts.

## Introduction

In the last few decades, an increasing number of talent identification and development (TID) programs in sports have been installed by professional sports clubs, commercial agencies and national governing institutions. All have the goal of identifying talented young athletes as early as possible in the hope of laying the foundations for superior senior performance and success in the long term [1]. Although the increase in early TID is likely the result of several factors (e.g., increased professionalization of elite sport, greater focus on organized sport during childhood), this process is also supported by research highlighting the value of ‘deliberate practice’ as an essential element of long-term development [2, 3] as well as by studies suggesting the 5-year period before pubertal growth is a sensitive period for the acquisition of motor skills [4–6]. However, there are many open questions regarding TID in young and developing players as indicated by the limited predictive value and questionable validity of TID programs in general [7–10].

In connection with the increase in TID programs, scientific research in the area of talent in sport and its related aspects has intensified. Gagné’s [11] Differentiated Model of Giftedness and Talent (DMGT) sees talent development as the “transformation of outstanding natural abilities (called [mental and physical] gifts) into outstanding knowledge and skills (called [competencies or] talents)” [11], while intrapersonal (e.g., motivation) and environmental catalysts (e.g., family) moderate this process. Discussing Gagné’s model in combination with other work and concepts in the field, Baker et al. [12] recently presented their starting point for a conceptualization of talent in sport. While Gagné defines a talent as a superb quality or type of a skill (or knowledge), Baker et al. see it as “that component of development that is present at birth differentiating it from skills, which reflect learned behaviors that may be confounded by talent” [12]. That is, the authors see a superb skill or performance as a consequence of talent and not as the talent itself, further specifying talent as “innate (i.e., originating in biological elements present at birth), multi-dimensional (i.e., consisting of capacities from a range of broad cognitive, physical, and psychological categories), emergenic (i.e., involving interactions among factors that combine multiplicatively), dynamic (i.e., evolving across developmental time due to interactions with environments and random gene expression) and symbiotic (i.e., cultural and social factors will determine the ultimate value of an individual’s talent)” [12]. This multidimensional, individual and dynamic character of talent has also been emphasized by other studies [13, 14], and forms the basis for how talent is positioned in the present review.

As TID decisions in young age groups are often based on singular talent scouting events or camps, a large number of factors (e.g., deciding coach/scout, athlete performance, opponent performance) have to coincide for young players to be selected into a TID program [15, 16], where they can further develop their skills benefiting from the system’s multifaceted resources (e.g., personal, organizational, financial). This early entry into the TID system and the associated benefits appears to be a crucial factor in talent development in many sports, especially when the environment outside the professionalized institutions is underdeveloped or non-existent in smaller, financially less resourced sports (e.g., female sports or marginalized sports such as climbing).

During the selection process, regardless of whether it is based on a singular event or a more longitudinal talent analysis, the sport-specific skill or performance level plays a crucial role and is dependent on the correct range of social, anthropometrical and physical/physiological factors as well as cognitive/psychological, tactical and technical skills [17, 18]. Previous research has been largely focused on the assessment of anthropometrical (e.g., height, wingspan) and physical/physiological performances (e.g., endurance, speed, agility, strength) which can be strongly influenced by differences in maturation and growth processes as well as different learning rates (e.g., leading to ‘relative age effects’ [19–22]), emphasizing the dynamic and individual nature of both performance and talent. This must always be considered when assessing both performance and talent determinants in developing athletes. While the assessment of anthropometrical and physical/physiological variables is understandable given their undeniable importance in many sports as well as their rather simple, proven and convenient assessment methods, the focus on these factors and the neglect of other determinants appears striking and leaves great potential untapped. In particular, technical skills seem to be essential given the highly demanding and specialized proficiencies required for high-quality performance in various sports [23, 24] even during early phases of development [25]. A few studies assessing sport-specific technical skills using a multidimensional approach found these skills predicted later performance in a range of sports, such as dribbling tests in field hockey [26], swimming performance and ball handling skills in water polo [27], the Loughborough Soccer Passing Test and dribbling, passing, shooting and ball control skills in soccer [28], and a slalom dribbling test in handball [29]. This research highlights the importance of technical skills within a multidimensional skill set. This skill set is not only dynamic and individual during the development of athletes, but also commonly unique to the sport and its role- and position-specific demands. In this context, an athlete showing inferiority in one skill area might be able to make up for it with superior skill in another area, as the skill requirements vary across different positions. This position specificity in combination with frequently used group comparisons (e.g., selected vs. non-selected, elite vs. non-elite), where performance/scores of all players of a team or group are combined, neglects differences across roles and positions and may lead to methodological problems and/or inconclusive results. Moreover, one has to consider how position-specific demands evolve as sports and strategies change, as well as the more general and less position-specific skills development in younger age groups where coaches and development policies aim to develop ‘all-rounders’ with a wide range of fundamental technical skills (i.e., not specialized players to fit into specific roles).

Furthermore, it is important to realize the interaction of technical skills with tactical skills (e.g., game-reading, anticipation, decision-making) as the successful execution of a technical–tactical strategy is always dependent on both skill types. The optimal automation of technical skills entails better possibilities for a player to execute tactical strategies as this automation frees up attentional resources that can be devoted to tactical and other objectives [30, 31].

Other research suggests the assessment of sport-specific technical skills is an important element of effective TID. It appears that sport-specific technical skills tests have the capacity to discriminate between low and high performance during pre-adolescence and adolescence (10–16 years of age) and to better predict future performance compared to other indicators [32–34]. However, technical skills can be operationalized and assessed in different ways (e.g., focusing on the outcome or the technique) and it is not clear which approach is best for TID across sports [35]. On the one hand, technical skills can be easily assessed by measuring the time, speed and/or accuracy on a sport-specific task (e.g., target goal kick in soccer or ball speed in baseball pitching; that is, measuring the outcome or result of the movement). On the other hand, more advanced assessment methods can provide valid and reliable electromyographic, kinematic and kinetic data of the human body during movement (i.e., measuring the movement technique being defined as describing “[…] the relative position and orientation of body segments as they change during the performance of a sport task to perform that task effectively” [35].) for the evaluation of technical skills within TID [25, 36].

The findings described above emphasize the crucial role of sport-specific technical skills in sports performance and accordingly in TID. Until now, to our knowledge, there has been no systematic overview on the assessment of sport-specific technical skills in the field of TID. An overview of both the applied assessment methods and the related findings is needed to improve existing approaches to TID as well as to develop new approaches to further exploration.

The aim of this systematic review was to provide a summary of studies assessing sport-specific technical skills, their specific assessment methods in more detail (analyzed by their method type and their method set-up; see Sect. 3.3 for details), and their findings in the context of TID research. Based on this state-of-the-science review, evidence-based suggestions are derived to guide future work in the field.

## Methods

### Search Design, Inclusion and Exclusion Criteria

This systematic review followed the guidelines of the Preferred Reporting Items for Systematic Reviews and Meta-Analyses (PRISMA) statement [37]. Searches were conducted in the electronic databases PubMed, Web of Knowledge, SPORTDiscus, SURF and Scopus, and were limited to peer-reviewed journal publications of original studies published in English between January 1990 and November 2019 (date of search: November 22, 2019). Search terms were adjusted to the settings and limitations of the respective database and covered the four areas of sport, technique, talent and youth:

- Sport coverage. Studies must cover a sport.
  *Search terms*: (sport* OR running OR “figure skating” OR diving OR soccer OR volleyball OR basketball OR handball OR football OR rugby OR “water polo” OR golf OR hockey OR korfball OR cricket OR baseball OR softball OR “table tennis” OR tennis OR badminton OR squash OR “weight lifting” OR ski OR skiing OR snowboard* OR swim* OR sprint* OR “long jump*” OR “high jump*” OR hurdl* OR javelin* OR discus* OR shot-put* OR pole OR cycling OR gymnastic* OR lacrosse OR skating OR wrestling) AND
- Technique assessment. The study must include an assessment of sport-specific technical skills.
  *Search terms*: (techni*) AND (test* OR measur* OR examin* OR assess* OR evaluat*) AND
- Talent assessment. Studies must contain a skill and/or performance level assessment. That is, they must conduct a group comparison (e.g., mean differences in elite vs. sub-elite) or relate the technical skills to (future) achievements (e.g., tournament or championship ranking).
  *Search terms*: (aptitud* OR talent* OR abilit* OR expert* OR gift* OR endowment OR excellen* OR success* OR perform* OR development OR identification) AND
- Youth coverage. Only studies investigating pre-adult (≤ 18 years) subjects were considered for this review.
  *Search terms*: (child* OR adolescen* OR boy* OR girl* OR youth* OR teen* OR young* OR puberty OR kid* OR junior* OR cadet* OR pupil* OR teen*).

After deleting duplicates and obtaining titles and abstracts, three authors (IF, JS, TK) independently screened the results based on the inclusion criteria above. Articles were excluded from the review if they did not represent journal publications of original studies (e.g., reviews, commentaries, or book chapters), handled general motor abilities and their assessment (e.g., KörperkoordinationsTest für Kinder), and/or did not relate technical skills to distinguishing between skill levels or predicting future performance. In cases where the titles and abstracts did not yield sufficient information to decide on inclusion, full-text articles were consulted. After this first set of articles was determined, reference lists of all articles still in the sample were checked for additional studies to be included based on the criteria above. This final list of articles was then included in both a quality check and the data synthesis.

### Quality Check

The methodological quality of all included articles was evaluated using a modified checklist based on the STROBE (Strengthening the Reporting of Observational Studies in Epidemiology) Statement [38] and its adaptations by Smith et al. [39]. Although the STROBE Statement’s checklist explicitly was not developed as an instrument to evaluate the quality of observational research, it includes a number of key components of high-quality research and is frequently used for quality evaluations within articles recently published in Sports Medicine (e.g., [39–41]). Following the goal of providing “guidance on how to report observational research well” [38], the STROBE Initiative developed an evaluation solution for the cohort and cross-sectional study designs that are highly prevalent in sports science. Accordingly, the checklist allowed for an evaluation of the research assessed in this systematic review that is at least as good as other common evaluation instruments (e.g. the Downs and Black checklist [42]).

The applied checklist included a total of 16 items assessing the articles’ overall quality based on a score of ‘0’ for missing or insufficient and ‘1’ for presented and sufficient information. After every item was rated independently by two researchers (IF, TK) and consensus was reached through discussion, the overall score for each article was calculated by summing the ratings of all items and transforming this overall score into categories of low, moderate and high quality based on scores of < 10, 10–13 and 14–16 [39], respectively.

### Data Extraction

Study characteristics were manually extracted into custom Excel workbooks (Microsoft Excel 2019, Microsoft Corporation, Redmond, Washington, USA) for all studies included in the review. The dataset included information on authors’ names, the journal and publication year, the sample’s country of origin, the sample size, the sport investigated, the sample’s performance level and the respective terminology applied, the participants’ age and sex, the applied assessment methods (including the method type and the method set-up, see Sect. 3.3 for a detailed description), and finally, the study’s findings.

Subsequently, information on the studies’ quality (see Sect. 2.2) and findings was combined to rate the level of evidence for every assessment method based on the rating system presented in Table 1. Each assessment method’s level of evidence represents the empirical evidence confirming (+) or rejecting (−) this method’s capability to discriminate groups of different skill/performance levels, explain past performance and/or predict future performance.

**Table 1:** Level of evidence ratings

Level of evidence	Definition	Rating
Conflicting	Conflicting results (< 2:1 ratio) between studies finding (no) discriminatory, explanatory and/or predictive effects	+/−
Limited	One study of high OR two studies of moderate quality find (no) discriminatory, explanatory and/or predictive effects	+ (−)
Moderate	Two studies of high OR three studies of moderate quality find (no) discriminatory, explanatory and/or predictive effects	++ (−)
Strong	At least three studies of high OR at least five studies of moderate quality find (no) discriminatory, explanatory and/or predictive effects	+++ (−)

## Results

**Fig. 1:**
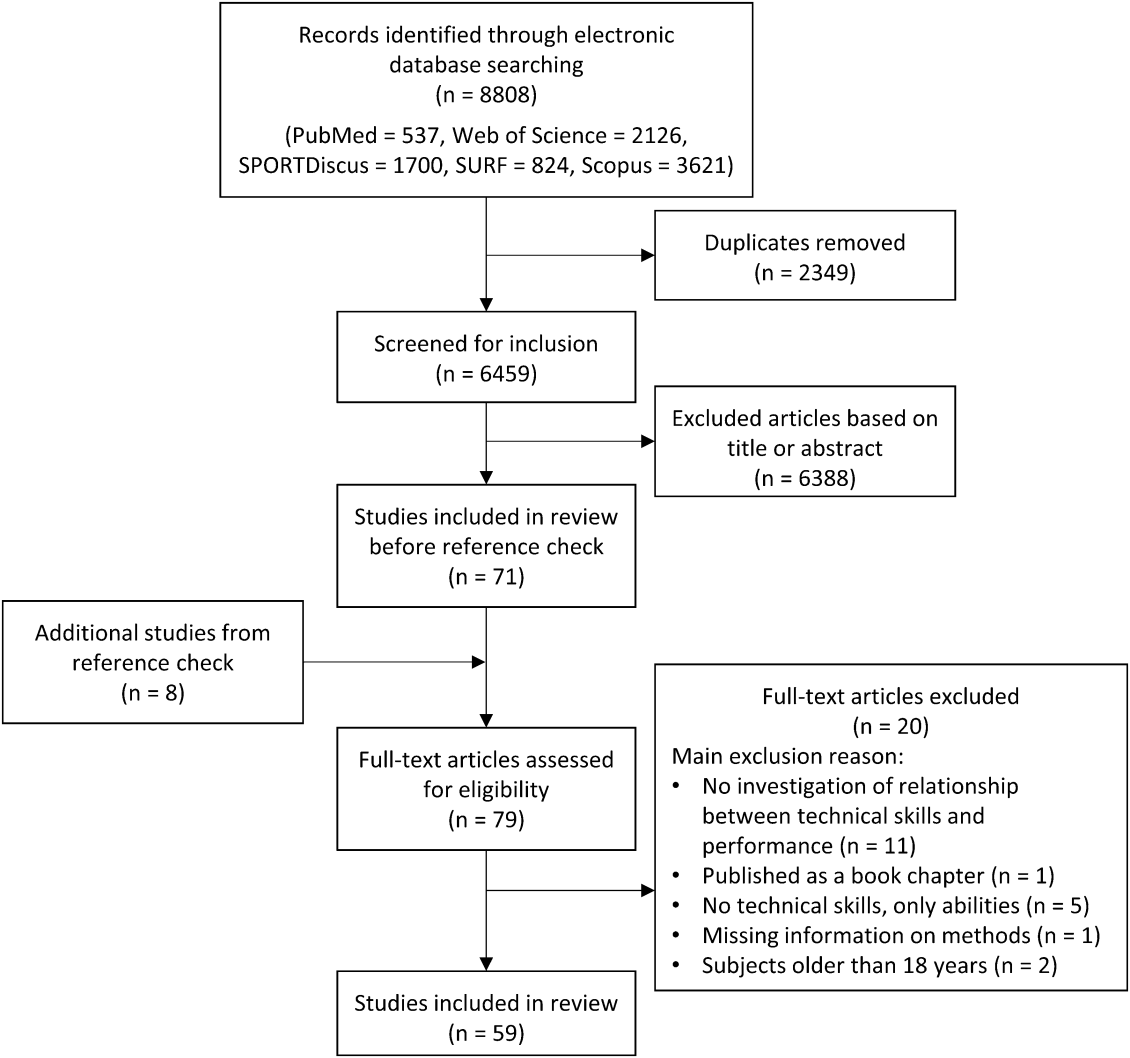
Flow diagram displaying the search’s work flow

The systematic database searches resulted in 8808 studies (see flow chart, Fig. 1). After removing duplicates (*n *= 2349) and excluding studies based on their title and abstract (*n *= 6388), the full text of 71 studies were reviewed. Examining the reference lists of those papers resulted in eight additional articles. After thorough assessment, 20 of the 79 articles were removed as they were missing either the relationship of technical skills and skill/performance level (*n *= 11) or detailed information on the methods (*n *= 1), assessed only technical abilities instead of sport-specific skills (*n *= 5), were published as a book chapter (*n *= 1) or investigated subjects older than 18 years (*n *= 2). Thus, a final number of 59 articles were analyzed for the qualitative synthesis.

### Quality Check

After independent rating, the two researchers (IF, TK) reached a sufficient agreement rate of 96% before differences were discussed and a consensus was reached for all items. The main differences between researcher ratings were for the two items ‘Handling missing information’ and ‘Providing effect sizes’. The quality check resulted in an average quality score of 14.42 (± 0.99) for all articles with the lowest scoring articles receiving scores of 12 and the best quality article having a perfect score of 16. Ten articles showed moderate quality and the remaining studies were rated as high quality (see Table 2). The largest deficits were found for the items ‘Study setting information’ (32/59 studies), ‘Study limitations’ (39/59 studies), ‘Providing effect sizes’ (41/59 studies), and ‘Participant information’ (42/59 studies).

**Table 2:** Characteristics of studies included in the systematic review

Study details					Participants			Methodological details			
Study	Design	Quality check	Sport	Country (ISO code)	Sex	*N*	Age (years)	Method type^a^	Method set-up^b^	Assessed sport-specific technical skills	Findings
Abdullah et al. [50]	CS	Moderate	Soccer	MYS	m	184	15.2 ± 2	OR	exp.	Slalom dribbling, passing, shooting	Technical skills discriminate players from different performance levels
Aquino et al. [51]	CS	High	Soccer	BRA	?	66	16.7 ± 0.4	OR	exp.	Shooting, ball control, slalom dribbling	Technical skills discriminate selected from unselected players
Archer et al. [44]	CS	High	Soccer	GBR	m	22	4.1 ± 0.7	OR	exp.	Straight dribbling, slalom dribbling	Technical skills discriminate players from different performance levels
Baiget et al. [52]	CS	High	Tennis	SPA	m	38	18.2 ± 1.3	OR	exp.	Stroking	Technical skills discriminate players from different performance levels
Bekris et al. [53]	CS	High	Soccer	GRC	?	48	16.7 ± 0.4	OR	exp.	Straight dribbling, slalom dribbling + dual task	Technical skills discriminate players from different performance levels
Bennett et al. [54]	CS	High	Soccer	AUS	m	55	13.3 ± 1.2	OR	comp.	Attempted/completed dribbles, passes, touches, shots, and Total actions	Technical skills discriminate players from different performance levels
Cripps et al. [55]	CS	Moderate	Australian Football	AUS	?	50	15.6 ± 0.4	OR	exp.	Coach evaluation of handballing and kicking	Technical skills discriminate non- vs. talent-identified players
Dardouri et al. [56]	CS	High	Soccer	TUN	m	92	14.2 ± 0.6	OR	exp.	Slalom dribbling, skill index (slalom dribbling/slalom sprinting without ball)	Technical skills discriminate players from different performance levels
Deprez et al. [57]	LT	High	Soccer	BEL	m	388	U10–U17	OR	exp.	UGent dribbling test, ball control	Technical skills discriminate dropouts from club players in specific age groups
D’Ercole et al. [58]	CS	Moderate	Water Polo	ITA	m	18	17.4 ± 0.47	OR	exp.	Straight swimming, straight dribbling, slalom dribbling, dual task condition	Technical skills discriminate players from different performance levels
di Cagno et al. [59]	CS	High	Rhythmic Gymnastics	ITA	f	25	14.7 ± 2.2	OR	exp.	Three jumping tests	Technical skills did not discriminate players from different performance levels
di Cagno et al. [60]	CS	High	Rhythmic Gymnastics	ITA	f	100	13.3 ± 0.5	TR	exp.	Four jumping tests	Technical skills are significantly correlated with rankings
Elferink-Gemser et al. [26]	LT	High	Field Hockey	NLD	both	63 m, 63 f	U12–U14	OR	exp.	Shuttle and slalom dribbling	Technical skills discriminate players from different performance levels
Elferink-Gemser et al. [61]	CS	High	Field Hockey	NLD	both	63 m, 63 f	13.9 ± 1.3	OR	exp.	Shuttle dribbling, slalom dribbling	Technical skills discriminate players from different performance levels
Falk et al. [27]	LT	High	Water Polo	ISR	m	24	12–16	OR	exp.	Swimming, dribbling, throwing	Technical skills discriminate selected from unselected players
Fenner et al. [62]	CS	High	Soccer	GBR	?	16	10.6 ± 0.3	TR	exp.	Coach evaluation for SSGs	Technical skills correlate with winning in SSGs
Figueiredo et al. [63]	CS	High	Soccer	PRT	m	159	11.82 ± 0.54	OR	exp.	Ball control, dribbling, shooting, passing	Technical skills discriminate players from different performance levels
French et al. [45]	CS	Moderate	Baseball	USA	both	130 m, 2 f	7–10	OR	both	Throwing, batting, catching	Technical skills discriminate players from different performance levels
Gabbett & Georgieff [64]	CS	High	Volleyball	AUS	both	14 m, 16 f	15.5 ± 1	TR	exp.	Spiking, passing, setting, serving	Technical skills discriminate players from different performance levels
Gabbett et al. [65]	CS	Moderate	Volleyball	AUS	?	28	15.5 ± 1	both	exp.	Spiking, passing, setting, serving	Technical skills discriminate selected from unselected players
Guimarães et al. [66]	CS	High	Basketball	PRT	m	150	13.3 ± 0.7	OR	exp.	Shooting, passing, dribbling, defensive movement	Technical skills discriminate selected from unselected players
Hendry et al. [67]	LT	High	Soccer	GBR	m	102	13–20	TR	exp.	Passing, dribbling, shooting or kicking	Technical skills discriminate different performance levels
Höner et al. [46]	CS	High	Soccer	GER	m	68,158	U12–U15	both	exp.	Slalom dribbling, ball control, passing, shooting	Technical skills discriminate different performance levels
Höner & Votteler [48]	LT	High	Soccer	GER	m	22,843	11.4 ± 0.3	both	exp.	Slalom dribbling, ball control/passing, shooting	Technical skills discriminate different performance levels
Höner et al. [47]	LT	High	Soccer	GER	?	14,178	U12–U15	both	exp.	Slalom dribbling, ball control/passing, shooting	Technical skills discriminate different performance levels
Höner et al. [68]	LT	High	Soccer	GER	f	499	11.4 ± 0.3	both	exp.	Slalom dribbling, ball control/passing, shooting	Technical skills discriminate different performance levels
Huijgen et al. [43]	Quasi-LT	High	Soccer	NLD	?	270	U12–U19	OR	exp.	Loughborough Soccer Passing Test	Technical skills discriminate selected from unselected players
Huijgen et al. [28]	CS	High	Soccer	NLD	?	113	17.1 ± 0.7	OR	exp.	Shuttle dribbling, slalom dribbling	Technical skills discriminate selected from unselected players
Huijgen et al. [69]	LT	High	Soccer	NLD	?	131	U12–U19	OR	exp.	Shuttle Dribble Test	Technical skills discriminate different performance levels
Keller et al. [70]	CS	High	Soccer	AUS	m	62	17 ± 0.61	OR	exp.	Loughborough Soccer Passing Test, long passing test, shooting, slalom dribbling	Technical skills discriminate different performance levels
Kolman et al. [71]	CS	High	Tennis	NLD	m	32	13.4 ± 0.5	OR	exp.	Dutch Technical-Tactical Tennis Test	Technical skills discriminate different performance levels
Le Moal et al. [72]	CS	Moderate	Soccer	FRA	m	87	15.1 ± 0.5	OR	exp.	Loughborough Soccer Passing Test	Technical skills discriminate players from different performance levels
Leyhr et al. [49]	LT	High	Soccer	GER	m	1134	U12–U15	OR	exp.	Slalom dribbling, ball control, shooting	Technical skills discriminate players from different performance levels
Lidor et al. [29]	LT	High	Handball	ISR	both	279 m, 126 f	12–14	OR	exp.	Slalom dribbling	Technical skills discriminate selected from unselected players
Lidor et al. [73]	LT	High	Volleyball	ISR	m	15	16–18	OR	exp.	Service accuracy	Technical skills did not discriminate players from different performance levels
Maszczyk et al. [74]	CS	High	Swimming	POL	?	189	12 ± 0.5	OR	comp.	Swimming technique analysis	Technical skills help with performance prediction
Naisidou et al. [75]	CS	High	Handball	GRE	f	91	13 ± 0.5	OR	exp.	Ball throwing velocity, slalom dribbling, triangle defense	Technical skills discriminate players from different performance levels
Rađa et al. [76]	CS	High	Soccer	CRO	m	119	16.2 ± 1.3	OR	exp.	Shooting	Technical skills discriminate players from different performance levels
Re et al. [77]	CS	High	Soccer	BRA	m	60	14 ± 0.93	both	both	Kicking ball speed, passing, in-game technical action	Technical skills did not discriminate starting from non-starting players
Re et al. [78]	CS	Moderate	Soccer	BRA	m	49	16.9 ± 0.5	both	exp.	Kinematic analysis of kicking, slalom dribbling	Technical skills discriminate players from different performance levels
Rebelo et al. [79]	CS	High	Soccer	PRT	m	180	18.1 ± 0.6	OR	exp.	UGent dribbling test, ball control	Technical skills discriminate players from different performance levels
Rebelo-Gonçalves et al. [80]	CS	Moderate	Soccer	PTR	m	40	14.5 ± 1.6	OR	exp.	Sprint-Keeper test (S-Keeper), Lateral Shuffle-Keeper test (LS-Keeper)	Technical skills discriminate players from different performance levels
Reilly et al. [81]	CS	Moderate	Soccer	GBR	m	31	16.4	OR	exp.	Shooting, slalom dribbling	Technical skills discriminate players from different performance levels
Rikberg & Raudsepp [82]	CS	High	Volleyball	EST	m	66	16.7 ± 0.7	both	exp.	Spiking, passing, setting, serving	Technical skills discriminate selected from unselected players
Saavedra et al. [83]	CS	High	Swimming	ESP	both	66 m, 67 f	12.6 ± 0.6	both	comp.	Swimming technique analysis	Technical skills discriminate players from different performance levels
Saward et al. [84]	LT	High	Soccer	GBR	m	126	U12–U18	OR	comp.	Frequencies of successful passes, on-target shots, dribbles, crosses, clearances, tackles/blocks/interceptions	Technical skills discriminate selected from unselected players
Silva et al. [85]	CS	High	Swimming	POR	both	65 m, 73 f	14.5 ± 0.4	TR	comp.	Semi qualitative swimming technical evaluation	Technical skills can help with performance prediction
Tangalos et al. [86]	CS	High	Australian Football	AUS	m	156	10–15	TR	exp.	Kicking, marking, handballing	Technical skills correlate with match performance.
Tribolet et al. [87]	CS	High	Australian Football	AUS	m	277	U13–U15	TR	exp.	Kicking, marking, handballing	Technical skills discriminate selected from unselected players
Vaeyens et al. [88]	CS	High	Soccer	BEL	?	232	U13–U16	OR	exp.	Slalom dribbling, passing, shooting, juggling	Technical skills discriminate players from different performance levels
Waldron & Murphy [89]	CS	Moderate	Soccer	GBR	?	31	14.1 ± 0.3	OR	both	Frequency of un-/successful passes, ball retentions and tackles; slalom dribbling, passing	Technical skills discriminate players from different performance levels
Waldron et al. [90]	CS	High	Rugby	GBR	?	57	U15–U17	OR	comp.	Frequency of un-/successful actions: carries, tackles	Technical skills did not discriminate players from different performance levels
Wilson et al. [91]	CS	High	Soccer	BRA	m	21	17.2 ± 1.1	both	exp.	Turn dribbling, coach rankings of defense skills in 1 vs. 1 competition	Technical skills help to detect defensively talented players
Woods et al. [92]	CS	High	Australian Football	AUS	?	55	U18	OR	comp.	Total disposals, marks, contested possessions, inside and rebound 50s	Technical skills are associated with better draft position
Woods et al. [93]	CS	High	Australian Football	AUS	?	84	17.5 ± 0.45	OR	exp.	Kicking, handballing	Technical skills discriminate selected from unselected players
Woods et al. [94]	CS	High	Australian Football	AUS	?	65	17.8 ± 0.5	OR	comp.	Total disposals, marks, contested possessions, inside and rebound 50s	Technical skills are associated with better draft position
Woods et al. [95]	CS	High	Australian Football	AUS	?	50	17.6 ± 0.55	OR	exp.	Kicking, handballing	Technical skills discriminate players from different performance levels
Zibung et al. [96]	LT	High	Soccer	CHE	m	104	U13–U16	OR	exp.	Slalom dribbling, passing, juggling	Technical skills are associated with better selection, but can be compensated
Zuber et al. [97]	LT	High	Soccer	CHE	m	119	U13–U16	OR	exp.	Slalom dribbling, ball control and passing, juggling	Technical skills are associated with better selection, but can be compensated

*CS* cross-sectional study design, *LT* longitudinal study design, *?* information not provided, *m* male, *f* female, *U* under, *OR* outcome-related, *TR* technique-related, *exp.* experimental, *comp.* competition, *SSGs* small-sided games

^a^‘Technique-related’ or ‘outcome-related’ for process or outcome focus, respectively

^b^‘Experimental’ or ‘competition’ for experiment or competition assessment of ecological validity, respectively

### Descriptive Results

Most articles were published since 2009 (*n *= 47) with a high increase since 2016 (*n *= 27); the oldest included article was from 1995; while all others were published after 2000. Almost two-thirds of the studies (*n *= 38) were from European countries; while the rest were conducted in Australia (*n *= 11), Brazil (*n *= 4), Israel (*n *= 3), Tunisia (*n* = 1), Malaysia (*n* = 1) and the USA (*n* = 1). The vast majority (*n *= 44) of studies used cross-sectional observations with 14 studies using a longitudinal design and one study applying a quasi-longitudinal approach [43]. The studies used a range of terminologies to describe the skill/performance level (e.g., non-talented vs. talented, novice vs. expert, non-elite vs. elite). Besides Archer et al. [44] and French et al. [45] dealing with young children (4.1 ± 0.7 and 7–10 years, respectively), all other studies examined samples between the ages of 10 and 18. Additionally, most samples were male (*n *= 31) compared to female only (*n* = 4) and mixed samples (*n *= 7). Seventeen studies did not provide information on the participants’ sex. Excluding four studies with extraordinary large sample sizes (*n *= 68,158, 14,178, 22,843 and 1134) [46–49] for this calculation, the mean sample size was *n *= 111 ± 100 subjects. The most commonly examined sports were soccer (*n *= 32) followed by Australian Football (*n *= 7) and volleyball (*n *= 4). The remaining studies included swimming (*n *= 3), field hockey (*n *= 2), rhythmic gymnastics (*n *= 2), water polo (*n *= 2), handball (*n *= 2), tennis (*n *= 2), rugby (*n *= 1), basketball (*n *= 1) and baseball (*n *= 1).

### Assessment Methods for Technical Skills

In this review, the assessments are categorized based on method type and set-up. These two categories describe the different aspects of both the measured variables and the data acquisition’s environment for assessing the sport-specific technical skills.

Method type was divided into ‘technique-related’ or ‘outcome-related’ approaches. Here, ‘technique-related’ measurement methods followed a process- or technique-focused approach; while ‘outcome-related’ methods investigated technical skills based on an outcome focus. For example, a coach’s evaluation or a biomechanical analysis (e.g., kinematic analysis of joint angles) of a throwing task reflect a qualitative and quantitative [98], ‘technique-related’ measurement of throwing skill. On the other hand, the number of shots made in this throwing task is considered as an ‘outcome-related’ assessment of throwing skill. Based on that classification, far more studies incorporated ‘outcome-related’ (*n *= 42) assessment methods of technical skills compared to ‘technique-related’ (*n *= 7) although some studies used a mix of both (*n *= 10).

**Fig. 2:**
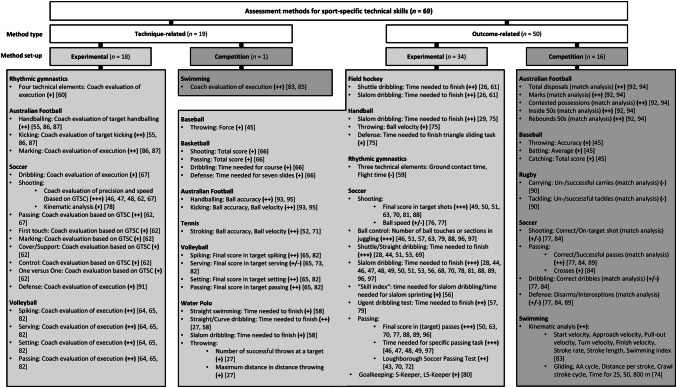
Overview of assessment methods included in the 59 studies; arranged by method type, method set-up, the different sports and the sport-specific technical skill assessed

To evaluate ecological validity and task representativeness, we examined the assessment method’s set-up and classified these as ‘experimental’ (i.e., experimental assessment of isolated actions assessing various variables, e.g., time needed for slalom course or shots made in shooting task) and/or ‘competition’ (i.e., video and notational analysis of competitions/matches assessing various variables, e.g., number of interceptions or successful passes) methods. Of the 59 studies, most applied ’experimental’ methods (*n* = 48) with only eight using ’competition’ measures; the remaining three studies used a combination of both assessment methods [77]. We then related this information to the different method types (‘outcome related’ vs. ‘technique related’) for all articles and found the 59 studies applied a total of 69 different assessment methods across sports (see Fig. 2).

Figure 2 also shows the wide range in assessment methods’ level of evidence (see Sect. 2.3 and Table 1) with seven ‘strong’, 28 ‘moderate’, 29 ‘limited’ and five ‘conflicting’ levels of evidence. For example, 18 (14 of ‘high’, four of ‘moderate’ quality) studies found a discriminatory, explanatory and/or predictive effect for the time needed to complete a slalom dribbling test in soccer, thus leading to a ‘strong’ and confirming (+++) level of evidence for this assessment method (see Fig. 2). In another example, only one study (of ‘high’ quality) found no such effect for the number of un-/successful carries in rugby, thus leading to a ‘limited’ and rejecting (−) level of evidence (see Fig. 2). Overall, most studies showed confirming results regarding the discriminatory, explanatory and/or predictive capabilities of sport-specific technical skills, while the level of evidence was mainly ‘limited’ to ‘moderate’, mostly because of a lack of studies applying the specific assessment methods.

### Discriminatory Value of Technical Skills Assessment

The most prevalent approaches in TID studies included in this review checked for differences (e.g., ANOVA-based approaches) in specific variables between athletes from different skill/performance levels (e.g., selected vs. unselected or elite vs. sub-elite). Other common approaches aimed to discriminate players based on those variables (e.g., discriminant analysis) or to relate those input variables to specific outcome variables (e.g., correlations or regression analysis). Of the 57 studies following these approaches, 53 found a significant discriminatory and/or explanatory value for the assessment of sport-specific technical skills. In the following, representative examples of these studies are presented and include data from basketball, Australian Football and soccer.

Conducting an ‘outcome-related’ and ‘experimental’ methods approach in basketball, significantly better shooting (Cohen’s *d* = 1.11), passing (*d* = 1.20), dribbling (*d* = − 1.62) and defense (*d* = − 1.05) skills were found for athletes selected to an elite regional team compared to non-selected athletes [66]. Also following an ‘outcome-related’ and ‘experimental’ methods approach, a study in Australian Football identified handball skills as being the most robust single measure (area under receiver operator curve = 76%, confidence interval = 62.5–89.5%) in a full logistic regression model to explain status (non-/state representation) [55]. In their study of young soccer players (U12–U15), Höner et al. [46] investigated technical skills applying an ‘experimental’ method set-up and a combination of both ‘technique-related’ and ‘outcome-related’ method types. They compared players from competence centers of the German Football Association (Deutscher Fußball Bund, DFB) with youth academy players from professional clubs expecting the latter to perform better. This was confirmed through significant results on tests of dribbling (‘outcome related’; Cohen’s *d* = 0.59–0.74) and juggling (‘outcome related’; Cohen’s *d* = 0.46–0.90) with ball control (‘outcome related’; Cohen’s *d* = 0.38–0.67) and shooting (‘technique related’; Cohen’s *d* = 0.08–0.34) skills showing smaller effects [46]. In another study of elite male youth soccer players, technical skills were collected following an ‘outcome-related’ and ‘competition’ method approach using notation analysis [84]. Analyzing the data with multilevel Poisson models, they found players retained by an academy performed more dribbles and retained defenders performed more tackles/blocks/interceptions compared to released players and defenders, respectively [84]. Other skills such as successful passes, on-target shots, crosses and clearances did not differ by playing status [84]. It is important to note that the authors considered the players' position to account for position-specific factors in their analysis and interpretation. In summary, the presented studies found sport-specific technical skills to be helpful in discriminating between groups of different skill/performance levels using various methodological approaches for data acquisition and analysis.

The four studies finding no discriminatory and/or explanatory value for the assessment of sport-specific technical skills followed different method type and set-up combinations in different sports. Thus, there is no clear tendency for a specific method type and set-up combination to be inferior. In their discussion, Di Cagno et al. [59] state that the high skill level in both groups (elite and sub-elite) probably led to similar results in the technical skills assessment in rhythmic gymnastics. Similarly, the homogeneity between groups probably also led to non-significant results comparing starters and non-starters in soccer [77] and in volleyball [73]. Furthermore, Waldron et al. [90] discussed their findings in the light of a small sample size (only one squad) and restricting the technical skills assessed to tackling and carrying skills while neglecting other crucial factors (e.g., passing, kicking). In summary, those authors emphasize the importance of population or sample as well as skill and method selection.

### Predictive Value of Technical Skills Assessment

In addition to the discriminating and explaining approaches above, two studies used a (quasi)-longitudinal approach to predict future swimming performance (e.g., in 1 year) with the help of neural networks and artificial intelligence procedures to compare these predictions with the reality (in this example, 1 year later) [74, 85]. For example, sport-specific technical skills predicted future 50-m (absolute error values of 20.39 s for *n* = 30) and 800-m (absolute error values of 4:11.96 s for *n* = 30) performances in swimming [74].

## Discussion

The aim of this systematic review was to provide an overview of studies assessing sport-specific technical skills and the specific methods/instruments that have been used for assessment, as well as to discuss these findings in the context of TID research. The assessment methods generally followed an ’outcome-related’ method type within an ’experimental’ method set-up. Most importantly, almost all studies found a discriminatory, explanatory and/or predictive value for the technical skill(s) being assessed. Notably, TID research has largely focused on cross-sectional study designs assessing male samples in soccer in European countries.

### Assessment Methods for Technical Skills

In this review, we established the two categories of *method type* and *method set-up* for the classification of different assessments. Our results showed the studies focused on ‘outcome-related’ method types and ‘experimental’ method set-ups, highlighting the outcome-focus and limited ecological validity and task representativeness of common assessment methods [25, 99]. Here, rarely used assessment methods incorporating ‘technique-related’ method types (e.g., biomechanical movement analysis) and/or ‘competition’ method set-ups (e.g. competition performance data) may offer possibilities; that said, the combination of both (i.e. biomechanical assessments in performance contexts) appears methodologically challenging. The potential of these methods is discussed in greater depth in Sect. 4.5.

### Discriminatory and Predictive Value of Technical Skills

Almost all studies included in this review found technical skills to be helpful when discriminating players from different skill/performance levels, explaining past performance or when predicting future performance. While this fact, on the one hand, emphasizes the crucial role of sport-specific technical skills in performance and talent, on the other hand, these results may have been affected by a publication bias. That is, there is a significant chance of studies assessing technical skills and finding no positive value for TID not being published (e.g., either due to bias on the part of the journal or the authors to publish null findings) [100]. This then leads to a biased interpretation of the overall field of available research. However, as presented and discussed above, we believe that sport-specific technical skills and their assessment have great potential, especially given the broad range of not-yet-employed assessment methods.

### Study Quality

The overall quality of studies included in this review was ‘moderate’ to ‘high’. If there were limitations, they were generally related to the methods, results and discussion. That is, some studies lacked crucial information on their sample (e.g., year the study was conducted or participants’ sex), gave no information on effect sizes and/or did not provide a sufficient discussion of their studies’ limitations or their findings’ generalizability. Although the overall quality was fairly high, these limitations reduce the studies’ validity to some degree. In addition to providing all necessary information, future studies should discuss the limitations and potential flaws in their work before debating the findings’ transferability to other contexts.

### Need for More Diverse Research

The descriptive results on the analyzed samples emphasized the need for more diverse research in TID as previously noted by Johnston et al. [17]. In particular, this relates to a greater diversity of sports, countries and sex/gender.

More than half of the studies (54%) analyzed samples in soccer, while only three other sports were investigated more than twice (7 × Australian Football, 4 × volleyball and 3 × swimming). The breadth and depth of work in soccer is not surprising as previous research and reviews have provided overviews on assessment methods in soccer (e.g. [101, 102]); however, the lack of research exploring technical skills in other sports was somewhat surprising. In addition, most of the studies in this review (64%) were conducted on samples from European nations, while the remaining studies included participants from Australia (19%), Brazil (7%), Israel (5%), Malaysia (2%) and the USA (2%). Furthermore, there was a clear over-representation of samples with male (64%) compared to studies including female participants (19%). This is noteworthy given the unique developmental and performance-related constraints of many female sports. Additionally, only two studies investigated athletes younger than 10 years of age, with the rest focusing on athletes between 10 and 18 years of age, which highlights the lack of knowledge in samples under the age of 10 years, despite the prevalence of TID activities (especially talent selection) in this age group. Collectively, the lack of information in many contexts (females, sports other than soccer, countries outside Europe, etc.), the often unique performance contexts across different sports, and the high investment in the field of TID in many countries, emphasize the importance of increased and more diverse research.

### Future Directions

Our results show there are only a few studies assessing sport-specific technical skills based on ‘technique-related’ method types and/or ‘competition’ method set-ups. In the future, biomechanical motion analysis and competition performance data could help to explore these assessment methods’ great potential.

#### Biomechanical Motion Analysis

Glazier [25] suggests that sports biomechanics—with its valid and reliable electromyographic, kinematic and kinetic data—could make valuable contributions to TID by advancing a more process-focused approach to gain a better understanding of the underlying patterns of coordination and control (i.e., technical skills) in more ecologically valid, sport-specific situations. He emphasizes the crucial roles of both control (i.e., absolute motion of a single body segment) and coordination (i.e., relative motion of body segments) in the execution and the analysis of movements. Here, for example, time-discrete kinematic variables (e.g., description of arm motion) could be used to identify associations with outcome variables (e.g., ball speed).

The potential of these methods increases further with the rise of innovative and steadily improving data collection and analysis tools. These types of technological advances will help to reduce the high organizational efforts of motion analysis. Camera systems with image recognition technology, markerless motion analysis systems or inertial measurement units (IMUs) are examples of improving technology tools for motion analysis that allow for more representative and ‘technique-related’ assessments with minimal athlete disruption. In addition to improvements and innovations in data collection tools, the fields of data processing and data analysis are evolving quickly (e.g., self-organizing maps, neural networks), enabling new ideas and approaches.

In one of the few studies following this approach, Zago et al. [103] examined dribbling skills in sub-elite players in soccer by conducting a 3D motion analysis during a slalom dribbling test. Afterwards, the data from this ‘technique-related’ method type were used to determine kinematic variables and cycle parameters. The researchers found differences between players with slower and faster dribbling test times for the foot-ball cadence, the mediolateral and vertical center of mass range of motion, the right stride cadence as well as the hip and the knee flexion range of motions. Although this study was not conducted in elite athletes and only tried to discriminate between dribbling test times and not overall skill/performance levels, the methodological approach illustrates the benefits and potential of ‘technique-related’ method types.

Sport-specific technical skills and their assessment as they relate to performance and talent evaluation eventually require some discussion of the need for a clear definition of an optimal or excellent technique for a given task. While this appears to be easier for constrained and closed movements (e.g., in gymnastics, cycling or rowing), it is harder to establish for open movements with many degrees of freedom (e.g., in basketball, soccer or baseball) [25], because in the latter, different control and coordination patterns can produce the same outcome parameters leading to the same result (e.g., two different shooting motions leading to the same ball trajectory and the same result in basketball). Furthermore, these ‘movement solutions’ (i.e., differing movements that lead to the same successful outcome) might be affected by individual organismic (e.g., anatomical) differences such as those are influenced by maturation and growth processes which end up altering technique due to changes in the body’s biomechanical limits (e.g., lever arms, moment of inertia and strength capacities) [104]. Thus, Glazier [25] emphasizes the need for “athlete-specific optimal techniques for different sports” [25] while at the same time stating this to be impossible at present. Here, although not issue free in both the modeling itself [105] and the following application (e.g., ‘intrinsic dynamics’ [104]), theoretical approaches such as computer simulation modeling [106] could at some point in time allow for more athlete-based approaches [107, 108].

However, linking important outcome parameters (e.g., ball rotation, ball speed, and ball launch angle in basketball throwing) to specific motion variables and investigating their relationships could already help, particularly with talent and technique development activities [25]. Furthermore, biomechanical assessment of technical skills in an ‘experimental’ method set-up could be connected with research on perception (e.g., quiet eye via eye tracking), as previous research has suggested that perception plays an important role in the execution of technical skills (e.g., advanced cues) [109, 110]; its inclusion would certainly increase the method’s ecological validity and representativeness.

In summary, despite a host of unique challenges, biomechanical motion analysis data could be used to deepen and extend relationships between sport-specific technical skills and performance. In addition, once developed and validated, these methods could be used for the evaluation of other, in terms of organization and data processing demands, less effortful (‘outcome related’) assessment methods (e.g., questionnaires or observation sheets).

#### Competition Performance Data

Another promising avenue for information on sport-specific technical skills is competition performance data (e.g., goals scored or successful passes). In many sports, quantifiable statistics from notational analyses [111] and activity profiling are commonly used as part of performance analytics, but are rarely used in TID. The few studies analyzing sport-specific technical skills with competition performance data (i.e., applying an ‘outcome-related’ method type and a ‘competition’ method set-up) found contested possessions/marks and inside 50s to be associated with better draft selection in Australian Football [92, 94]; while, number of dribbles or tackles/blocks/interceptions, dribbling speed and (un-)successful ball retentions were able to discriminate elite from sub-elite or selected from unselected players in soccer [84, 89]. Accordingly, assessing sport-specific technical skills quantitatively during competition seems to be a valuable and highly ecologically valid approach. However, these studies also noted that their data are affected by growth and maturity processes as less mature rugby players were not selected despite performing higher volumes of high-intensity running [90]. Although volumes of high-intensity running are not considered technical skills, the latter are probably also affected by maturity and relative age effects. Accordingly, early maturing soccer players performed higher numbers of tackles/blocks/interceptions [84]. As the understanding of the underlying relationships is limited to date, further research should examine a range of variables across different sports and their connection to talent and performance. Here, quantitative data could be combined with qualitative data (e.g., interviews targeting important variables/factors) to complete the profile using a mixed methods approach. Furthermore, learning from sports that utilize judging systems to evaluate the quality of technical skills (e.g., figure skating or artistic gymnastics) may be worthwhile as they feature ‘technique-related’ and/or ‘outcome-related’ method type in combination with a ‘competition’ method set-up.

Studies using competition performance data suggest some potential for improving our understanding of TID and the methods used for identification and selection. The discriminatory and predictive value of these data increases even more given the growing variety of analytical methods and computing power in combination with the development of new technical and tactical game performance statistics [112].

### Limitations

Despite the intriguing findings summarized above, there were some limitations to our systematic review. First, restricting our search to only English articles in five databases probably led to missing articles and knowledge published in other languages. Accordingly, future studies could complement our findings by integrating searches and articles in other languages. Second, our findings are likely influenced by an already discussed (see Sect. 4.2) publication bias towards positive results. As future studies should try to combine data for meta-analyses, a statistical evaluation of the publication bias should be included. Third, there might be reliable and valid assessment methods for technical skills in practice, but no studies checked for their scientific and practical value in TID so far and thus, they were not integrated into our review. Fourth, our approach to assess ecological validity via the method set-up as ’experimental’ or ’competition’ is rather simplistic and is meant to be only a first step in the direction of this classification. Future studies should apply a more differentiated system and consider evaluating the reciprocal effects of ecological validity and controlling variables in an experimental set-up. Fifth, despite most sports conducting their TID activities before adulthood, restricting our search to studies of samples younger or equal to 18 years of age may have neglected sports with a higher age of peak performance. Future research should consider this age of peak performance for certain sports. Sixth, looking at the distribution of sports assessed in the included articles, there is a range of sports with very different performance and skill requirements as represented by team-ball-sports like Australian Football, racquet sports like tennis or individual sports like swimming. In addition, the representation of various sports shows a significant skew towards soccer with minimal coverage of other sports. Thus, the results of the present review are most valid for TID in soccer and should be generalized and transferred to other sports with caution. However, given the similarly crucial role of sport-specific technical skills in many sports, this generalization and transfer appears legitimate at least for proposing hypotheses that need to be tested. Based on this review, analysis of sport-specific technical skills using ‘technique-related’ biomechanical assessment methods and performance data seems to be a promising direction for future research to add to the existing knowledge base.

## Conclusions

Our results emphasize that the assessment of sport-specific technical skills is an integral part of comprehensive, multidimensional and longitudinal approaches within TID contexts. Accordingly, they should be investigated to further extend existing approaches and develop new tools for the ‘technique-related’ assessment of sport-specific technical skills, particularly given the advancements in technologies associated with sports biomechanics and match analysis. Furthermore, following a ‘competition’ method set-up, the application of competition performance data should be expanded in the context of TID. In addition, the combination of assessment methods seems to offer a useful and more ecologically valid source of information for TID. Collectively, this research will be useful for both scientists developing new TID tools and coaches in the practice of TID as it improves our understanding of the value of technical skills across the athlete pathway, thereby improving the quality of TID decision-making, and ultimately leading to better sport performances.

## Data Availability

The datasets generated and/or analyzed during the current review are available from the corresponding author on reasonable request.
